# Chronic pain and cognitive dysfunction: clinical manifestations, underlying mechanisms, and emerging therapeutic strategies

**DOI:** 10.3389/fnins.2025.1641903

**Published:** 2025-10-21

**Authors:** Taowu Gong, Wanqiu Yu, Pengcheng Zhao, Zhenyu Wu, Yuhang Zhu, Qihai Gong, Zhaoqiong Zhu

**Affiliations:** ^1^Department of Anesthesiology, Affiliated Hospital of Zunyi Medical University, Zunyi, China; ^2^Key Laboratory of Brain Function and Brain Disease Prevention and Treatment of Guizhou Province, Zunyi, China; ^3^Department of Anesthesiology, The Frist People's Hospital of Zunyi, Zunyi, China; ^4^Early Clinical Research Ward, Affiliated Hospital of Zunyi Medical University, Zunyi, China; ^5^Key Laboratory of Basic Pharmacology of Ministry of Education and Joint International Research Laboratory of Ethnomedicine of Ministry of Education, Zunyi Medical University, Zunyi, China

**Keywords:** chronic pain, neuropathic pain, hippocampus, gut microbiota, learning and memory, cognitive dysfunction, therapeutic strategies

## Abstract

Chronic pain affects up to 60% of the population and not only impairs physical function but also leads to multidimensional neurocognitive deficits, including diminished attention, working memory impairment, and executive dysfunction. Clinical studies indicate that chronic pain induces gray matter atrophy in key brain regions, such as the prefrontal cortex and hippocampus, along with disrupted functional connectivity and other pathological alterations. Despite extensive research, the precise pathogenic mechanisms remain largely unclear, making this a central focus of current investigations. In this review, we examine the morphological and functional changes in these critical brain regions from an anatomical perspective. By integrating cellular and molecular insights, we elucidate the multi-level mechanisms underlying chronic pain-induced cognitive impairment. Furthermore, we summarize current therapeutic strategies, including pharmacological treatments, neuromodulation, and behavioral interventions, and discuss promising directions for future research. By synthesizing recent advances, this review aims to enhance understanding of the clinical manifestations and pathophysiology of chronic pain, thereby informing the development of more effective diagnostic and therapeutic approaches.

## 1 Introduction

Chronic pain is a multifaceted condition encompassing neurological, psychological, and social dimensions, and it has garnered increasing attention due to its association with cognitive dysfunction. The International Association for the Study of Pain (IASP) defines chronic pain as pain persisting or recurring for more than 3 months. It can manifest both as a symptom of other diseases and as an independent pathological entity (Mäntyselkä et al., 2003; Verhaak et al., 1998). Epidemiological studies indicate that up to 60% of the global population, across diverse age groups and socioeconomic backgrounds, is affected by chronic pain, resulting in substantial healthcare costs and societal burdens (Elliott et al., 1999; Sadlon et al., 2023; Zhao et al., 2023). Importantly, chronic pain frequently co-occurs with mood disorders, sleep disturbances, and cognitive impairments (Alotaibi et al., 2025). Patients commonly exhibit anxiety and a spectrum of cognitive deficits, including attentional impairments, reduced executive function, and slowed information processing, with the severity of cognitive decline positively correlating with pain intensity and duration (Rong et al., 2021; Rader et al., 2025). These observations underscore the urgent need to identify risk factors contributing to cognitive decline in chronic pain, thereby enabling timely preventive and interventional strategies.

Preclinical evidence consistently demonstrates that chronic pain adversely affects cognitive function, although the precise mechanisms remain incompletely understood (Viero et al., 2025; Liu et al., 2025). Current research suggests that the pathophysiology of chronic pain-related cognitive dysfunction is multidimensional, involving alterations in neural plasticity, neuroinflammation, neurotransmitter system imbalances, structural and functional brain changes, epigenetic modifications, and gut-brain axis dysregulation (Han et al., 2024a; Pak et al., 2024). Despite an increasing array of clinical interventions, treatment remains challenging. Analgesics including opioids may partially alleviate pain but often have limited efficacy in improving cognitive function and may even exacerbate memory deficits by impairing synaptic plasticity (Dick and Rashiq, 2007; Schiltenwolf et al., 2014). Non-pharmacological interventions are constrained by individual variability and incomplete mechanistic targeting, while emerging therapies aimed at glial cell modulation, epigenetic regulation, and gut-brain axis restoration remain largely preclinical, highlighting substantial translational barriers.

Clinically, cognitive dysfunction in chronic pain patients has profound implications. Impaired attention, memory, and executive function can hinder patients’ ability to accurately report symptoms, adhere to treatment regimens, and engage in self-management strategies, ultimately compromising pain control and rehabilitation outcomes. These deficits also exacerbate emotional distress, reduce social participation, and significantly diminish quality of life. Moreover, cognitive impairment may alter patients’ responsiveness to both pharmacological and non-pharmacological interventions, thereby influencing prognosis. Despite its high prevalence and impact, the interplay between chronic pain and cognition remains under-recognized in routine clinical practice, and mechanistic insights are fragmented across disciplines. This review aims to bridge these gaps by integrating preclinical and clinical evidence, delineating convergent biological pathways, and highlighting emerging therapeutic strategies targeting the shared mechanisms of pain and cognitive decline. This review comprehensively summarizes current progress in understanding chronic pain-induced cognitive dysfunction, explores potential therapeutic strategies and future research directions, and provides a theoretical basis for clinical diagnosis, treatment, and mechanistic investigations.

## 2 Cognitive dysfunction associated with chronic pain: clinical research

### 2.1 Current status of clinical research

The comorbidity of cognitive dysfunction with chronic pain has emerged as a critical focus in clinical research. Epidemiological studies report chronic pain prevalence rates ranging from 11 to 60% (Mäntyselkä et al., 2003; Sadlon et al., 2023). Clinical and preclinical evidence suggests that at least 50% of individuals with chronic pain exhibit cognitive impairments (Cohen et al., 2021), with the incidence of mild cognitive impairment (MCI) showing a dose-dependent relationship with pain severity (Jorge et al., 2009). A systematic review identified 53 tools used to assess cognitive function, 73.8% of which were neuropsychological assessment scales; however, no instruments are specifically tailored for patients with chronic pain (Ojeda et al., 2016). The use of diverse assessment tools may yield heterogeneous results, introducing systematic bias. Therefore, a unified cognitive assessment framework is urgently needed to elucidate the relationship between chronic pain and cognitive function.

Systematic reviews and meta-analyses consistently demonstrated that chronic pain substantially increases the risk of cognitive decline and dementia (Innes and Sambamoorthi, 2020; Yuan et al., 2023). Longitudinal cohort studies indicate that chronic pain is associated with accelerated cognitive deterioration and a higher likelihood of developing dementia (Whitlock et al., 2017; Rong et al., 2021). In middle-aged and older populations across six low-income countries, pain severity correlates with the incidence of MCI in a dose-dependent manner (Smith et al., 2023). Trajectories of pain and activity limitations are significantly linked to the rate of cognitive decline in older adults (He et al., 2024). Multiple cross-sectional studies report that chronic pain patients score significantly lower than healthy controls in memory, attention, executive function, and information processing speed (Oosterman et al., 2012; Higgins et al., 2018). Persistent pain has been shown to accelerate cognitive decline over a 10-year period (Whitlock et al., 2017), and pain intensity is significantly associated with cognitive dysfunction (Van der Leeuw et al., 2018). Some studies suggest that each additional two-year period of pain interference increases the risk of cognitive impairment by 21% (Bell T. et al., 2022). A bidirectional Mendelian randomization study confirmed a causal relationship between multi-site chronic pain and cognitive dysfunction, with no evidence of a reverse association (Guo et al., 2023). Nonetheless, a limited number of studies report divergent findings. High heterogeneity in study design, assessment tools, and sample characteristics currently precludes the establishment of a definitive causal link between chronic pain and cognitive decline (Zhang X, et al., 2021; Sadlon et al., 2023). The role of sex in chronic pain-related cognitive dysfunction remains debated (Segura-Jiménez et al., 2016; Zhang et al., 2024). Some evidence suggests that women may be particularly susceptible, potentially due to the modulatory effects of sex hormones (Roth et al., 2005; ter Horst et al., 2012). Estrogen, for instance, exerts neuroprotective effects by enhancing hippocampal synaptic transmission and inhibiting microglial activation, yet cyclical hormonal fluctuations may increase pain sensitivity and compete for cognitive resources, providing a biological basis for gender differences (Pozzi et al., 2006; Bartley and Fillingim, 2013). Further studies are needed to clarify phenotype-specific mechanisms underlying sex differences.

In conclusion, despite heterogeneity in methodologies and assessment tools, the majority of evidence supports a detrimental impact of chronic pain on cognitive function. Future research should aim to establish standardized diagnostic criteria for chronic pain and a unified cognitive assessment system, integrating neuroimaging techniques and biomarker analyses to clarify the underlying pathophysiological mechanisms linking chronic pain and cognitive impairment.

### 2.2 Clinical manifestations of cognitive dysfunction induced by chronic pain

Clinical evidence indicates that acute pain may exert protective effects, whereas chronic pain lacks such benefits and is consistently associated with cognitive impairments. Chronic pain affects multiple cognitive domains, including learning, memory, attention, information processing speed, working memory, long-term memory, and executive function (Phelps et al., 2021b; Phelps et al., 2021a; Zhou et al., 2022). Adolescents experiencing pain exhibit reduced cognitive performance compared to healthy peers (Jastrowski Mano et al., 2020), while older adults with chronic pain demonstrate more pronounced cognitive decline (Murata et al., 2017; Chen J. et al., 2023). These impairments are not uniform across pain conditions. Based on the clinical studies summarized in Table 1, patients with different types of chronic pain consistently exhibit cognitive deficits, although the affected domains vary. Fibromyalgia is primarily associated with deficits in divided attention (Moore et al., 2019), whereas osteoarthritis, particularly chronic hip osteoarthritis, is linked to domain-specific impairments involving short- and long-term memory, attention, and executive function (Kazim et al., 2023). Chronic low back pain is characterized by more widespread cognitive impairments, including deficits in attention, working memory, information processing speed, executive function, language, and visuospatial abilities (Corti et al., 2021). Overall, attention and executive dysfunction emerge as common features across multiple pain types, while the extent and pattern of memory, language, and visuospatial deficits differ depending on the underlying pain condition, suggesting that specific pain phenotypes may be associated with distinct cognitive impairment profiles.

**Table 1:** Clinical studies on cognitive impairment associated with chronic pain.

Pain type	Cognitive assessment tool	Cognitive domain	Key findings on chronic pain-cognition association	Reference
Chronic low back pain	WAIS-III, TMT, SDMT, Stroop, WCST, PMQ	Information processing speed, Working memory, Executive function, Short-term memory	Chronic low back pain patients exhibit impairments in working memory, cognitive flexibility, and information processing speed	(Ling et al., 2007; Schiltenwolf et al., 2014; Baker et al., 2018)
Chronic pancreatitis pain	Integneuro test battery	Psychological performance, Memory, Executive function	Patients with chronic pancreatitis pain demonstrate reduced scores across multiple cognitive domains, with the most pronounced deficits in psychological performance and executive function	(Jongsma et al., 2011)
Non-cancer chronic pain	MoCA, Stroop	Information processing speed, Attention	1. Patients with non-cancer chronic pain exhibit mild cognitive deficits. 2. Neuropsychological function in chronic pain patients resembles that of healthy controls.	(Ferreira et al., 2016)
Chronic pain	CASI, Stroop, WAIS, WCST, SCWT, WAIS	Short-term memory, Attention, Executive function	1. Patients with chronic pain and osteoarthritis exhibit poorer cognitive performance. 2. The pain group demonstrates significantly impaired performance in attention and executive function compared to controls	(Arévalo-Martínez et al., 2024; Wen et al., 2024)
Chronic pain	Stroop, LNS, WTAR, CVLT, EMQR, BAPM, RBANS, TMT	Memory, Attention, Executive function	1. Chronic pain patients significantly underperform healthy controls in attention and executive function. 2. Opioid-treated patients exhibit a further reduction in attention performance compared to non-opioid users	(Richards et al., 2018; van der Leeuw et al., 2018)
Chronic pain	MMSE, TMT, CERAD-Plus	Executive function, Memory performance	Patients with chronic pain and MCI exhibit significant cognitive impairments	(Lautenbacher et al., 2021)
Chronic low back pain	WAIS-IV, HVLT, BNT, JLO, HVOT	Executive function, Attention, Working memory, Language, Visuospatial ability	Patients with chronic low back pain demonstrated significantly poorer performance in attention, working memory, language, and visuospatial tasks compared to healthy controls	(Corti et al., 2021)
Chronic hip osteoarthritis pain	MMSE, RBMT, TMT, F-A-S test	Short-term and long-term memory, Attention, Executive function	Chronic hip osteoarthritis pain is associated with domain-specific cognitive impairments	(Kazim et al., 2023)
Chronic musculoskeletal pain	TMT, DSST	Executive function, Processing speed	Older adults with chronic musculoskeletal pain exhibit significantly impaired processing speed	(Murata et al., 2017)
Multisite chronic pain	MoCA	Executive function, Attention	Patients with multisite chronic pain demonstrate poorer cognitive performance in attention and executive function compared to controls	(Cardoso et al., 2021)
Fibromyalgia	n-back task, attention-switching task, divided attention task	Attention	Fibromyalgia patients show reduced performance in divided attention tasks	(Moore et al., 2019)

TMT, Trail Making Test; WAIS, Wechsler Adult Intelligence Scale; PMQ, Prospective Memory Ques-tionnaire; CASI, Cognitive Abilities Screening Instrument; WCST, Wisconsin Card Sorting Test; SCWT, Stroop Color and Word Test; BRIEF-A, Behavior Rating Inventory of Executive Function–Adult Version; SDMT, Symbol Digit Modalities Test; Stroop, The Stroop Color and Word Test; WTAR, Wechsler Test of Adult Reading; CVLT, California Verbal Learning Test; EMQR, Everyday Memory Questionnaire–Revised; LNS, Letter-Number Sequencing; BAPM, Brief Assessment of Pro-spective Memory; RBANS, Repeatable Battery for the Assessment of Neuropsychological Status; CERAD-Plus, Consortium to Establish a Registry for Alzheimer’s neuropsychologic battery; SCD, Subjective cognitive decline; BRFSS, Behavioral Risk Factor Surveillance System; HVLT, Hopkins Verbal Learning Test-Revised; BNT, Boston Naming Test; JLO, Judgment of Line Orientation; HVOT, Hooper Visual Organization Test; RBMT, Rivermead behavioral memory test; F-A-S test, Verbal fluency F-A-S test; DSST, Digit Symbol Substitution Test.

#### 2.2.1 Learning and long-term memory

Clinical studies have demonstrated that chronic pain elevates the risk of memory impairments and is associated with multidimensional deficits across cognitive domains (Innes and Sambamoorthi, 2020). Cognitive dysfunction is significantly more prevalent in chronic pain patients than in healthy populations, affecting attention, executive function, and learning and memory. Among these, learning and memory functions appear particularly susceptible to chronic pain, with pain persistence correlating with accelerated memory decline (Higgins et al., 2018).

Patients with fibromyalgia and osteoarthritis, two common subtypes of chronic pain, perform worse on delayed recall and working memory tasks compared to healthy controls (Apkarian et al., 2011). Meta-analyses further reveal small to moderate deficits in long-term memory among fibromyalgia patients relative to healthy adults (Bell et al., 2018). Persistent moderate to severe pain exhibits a dose–response relationship with subsequent memory decline (Rong et al., 2021). Pain subtypes also demonstrate domain-specific cognitive associations: osteoarthritis primarily affects visuospatial and executive functions, whereas fibromyalgia predominantly impairs working memory (Tiara and Fidiana, 2021). The severity and duration of pain are key determinants of cognitive outcomes. Patients with moderate to severe joint pain face a higher risk of memory decline than those with mild pain, and each additional year of pain duration is associated with progressive reductions in episodic memory scores (van der Leeuw et al., 2018; Horgas et al., 2022). Longitudinal studies indicate that individuals with persistent pain exceeding 6 months have a substantially increased risk of developing major memory impairments over a 10-year period, independent of confounding factors such as age and education (Whitlock et al., 2017). Collectively, these findings indicate that chronic pain significantly impairs learning and long-term memory, with pain intensity, subtype, and duration serving as critical factors informing clinical intervention strategies.

#### 2.2.2 Attention

Multiple clinical studies have confirmed a robust association between chronic pain and attentional dysfunction. Chronic pain impairs various aspects of attention, including sustained, selective, and divided attention (Bell et al., 2018). Acute experimentally induced pain and chronic pain affect attention differently: acute pain primarily reduces accuracy in n-back and attention-switching tasks, whereas chronic pain patients exhibit deficits in divided attention tasks (Moore et al., 2019). Evidence indicates that chronic pain disrupts performance on attention-demanding tasks (Higgins et al., 2018) and alters brain activity associated with attentional processing (Pinheiro et al., 2016). Clinically, many patients report difficulties concentrating, with some experiencing persistent attention deficits (Legrain et al., 2009). Impairments in attention may underlie the overall mild cognitive impairment observed in chronic pain populations (Ferreira et al., 2016). Prospective studies show that patients with knee osteoarthritis experience declines in short-term memory and attention, with the effects being more pronounced in those with chronic pain (Wen et al., 2024). In specific domains, such as selective and sustained attention, task performance efficiency is generally lower in chronic pain patients compared to healthy controls (Arévalo-Martínez et al., 2024). Chronic pain also impairs internal attention, thereby hindering creative thinking, and significantly reduces performance in attention-demanding tasks (Richards et al., 2018; Gubler et al., 2022). Collectively, these findings suggest that chronic pain exacerbates cognitive load by disrupting core attentional processes, such as information filtering, sustained focus, and multitasking, ultimately contributing to broader cognitive decline.

#### 2.2.3 Executive function

Cognitive flexibility, a critical component of executive function, is primarily mediated by the prefrontal cortex (PFC; Cowen et al., 2018). Accumulating evidence indicates that patients with chronic pain often exhibit mild to moderate impairments in executive function, with deficits in this domain being particularly pronounced (Berryman et al., 2014). In individuals with MCI, both executive function and memory are compromised, suggesting that pain may accelerate cognitive decline in this vulnerable population (Lautenbacher et al., 2021). Adolescents suffering from chronic musculoskeletal pain also show poorer executive function compared with age- and sex-matched healthy controls (Jastrowski Mano et al., 2020). Moreover, chronic pain patients receiving long-term opioid therapy demonstrate significant deficits in cognitive flexibility, highlighting the combined impact of pain and pharmacological treatment on executive function (Schiltenwolf et al., 2014). Notably, the duration of pain emerges as the strongest predictor of cognitive decline, with longer-lasting pain correlating with more severe impairments in cognitive flexibility (Jongsma et al., 2011; Cowen et al., 2018). Although overall cognitive dysfunction in chronic pain is generally mild, deficits in specific domains such as executive function are more prominent relative to healthy individuals (Richards et al., 2018; Arévalo-Martínez et al., 2024). These observations are consistent with findings in chronic low back pain, where impairments in executive function and working memory have also been reported (Baker et al., 2018; Richards et al., 2018; Corti et al., 2021). Collectively, current evidence suggests a mild to moderate association between chronic pain and executive function, underscoring the need for further studies to elucidate underlying mechanisms and develop targeted intervention strategies, including neuroimaging investigations.

#### 2.2.4 Short-term memory

Chronic pain is frequently associated with deficits in working memory, a core component of short-term memory, as well as other cognitive domains. The bidirectional relationship between pain and working memory impairment has been well documented (Higgins et al., 2018; Procento et al., 2021). Clinical studies consistently show that, compared with pain-free individuals, patients with chronic pain not only perform worse on working memory tasks but also report greater subjective deficits (Mazza et al., 2018; Rader et al., 2025). Longitudinal research in older adults indicates that persistent pain interference correlates with declines in overall cognitive function, particularly in immediate and delayed memory (Bell T. et al., 2022). Similarly, Ling et al. (2007) reported significant impairments in prospective memory among patients with chronic back pain relative to controls. Chronic low back pain and fibromyalgia patients also exhibit lower performance on working memory and short-term memory assessments compared with healthy populations (Bell et al., 2018; Corti et al., 2021) Disease-specific analyses reveal that individuals with hip osteoarthritis show notable reductions in short-term memory on neuropsychological testing (Kazim et al., 2023). Moreover, chronic pain patients undergoing long-term opioid therapy demonstrate even greater working memory impairments, suggesting that pharmacological factors may exacerbate cognitive deficits (Schiltenwolf et al., 2014). Collectively, these findings indicate that chronic pain exerts a substantial negative impact on short-term memory function.

#### 2.2.5 Information processing speed and mental flexibility

Cognitive dysfunction in chronic pain patients is reflected in standardized tests as slower reaction times and reduced information processing efficiency. Among cognitive domains, processing speed appears particularly vulnerable to the effects of pain, often more so than memory or reasoning (Bell T. R. et al., 2022). Studies demonstrate that, relative to healthy controls, individuals with chronic pain exhibit significant deficits in basic cognitive tasks, including visual attention, graph processing speed, visual scanning, and number sequencing (Schiltenwolf et al., 2014). Their information processing speed and mental flexibility are also markedly impaired (Ferreira et al., 2016). Prospective epidemiological evidence indicates that declines in processing speed among chronic pain patients occur independently of confounding factors such as age and education (Rouch et al., 2021). Cross-sectional analyses across different pain subtypes support these findings: patients with chronic low back pain show significant impairments in processing speed (Corti et al., 2021), whereas individuals with fibromyalgia demonstrate reduced information processing efficiency compared to healthy populations (Serrano et al., 2022). Community-dwelling older adults with chronic musculoskeletal pain similarly exhibit delayed processing speed (Murata et al., 2017). Cognitive deficits in chronic pain are often accompanied by impairments in delayed memory, problem-solving abilities, and altered psychological states. Importantly, effective interventions targeting pain can partially restore information processing efficiency and overall cognitive function, likely by reducing central nervous system (CNS) overload (Abd-Elsayed and Gyorfi, 2023). Collectively, current clinical evidence consistently supports the association between chronic pain and declines in neurocognitive performance, with core impairments predominantly involving processing speed, attention, and memory. These findings provide a theoretical basis for implementing cognitive-protective strategies in pain management.

### 2.3 Potential pathogenic mechanisms

Neuroimaging studies have demonstrated that chronic pain can induce both structural and functional remodeling in brain regions critical for cognitive function. Notably, reductions in gray matter volume within the medial prefrontal cortex (mPFC), dorsolateral prefrontal cortex (DLPFC), and hippocampus constitute core pathological substrates underlying cognitive impairments (Murata et al., 2017; Tan et al., 2022). These alterations are negatively correlated with both pain duration and advancing age, suggesting that chronic pain may accelerate brain atrophy and promote pathological aging processes (Geisser and Kratz, 2018). Large cohort analyses indicate that patients with multi-site chronic pain exhibit greater hippocampal volume reductions and faster cognitive decline compared to single-site pain sufferers and healthy controls, highlighting the cumulative neural damage associated with widespread pain (Zhao et al., 2023).

Subtype-specific analyses reveal heterogeneous patterns of brain remodeling. Patients with fibromyalgia show significantly reduced gray matter density in the cingulate gyrus, insula, and parahippocampal gyrus, which positively correlates with disease progression (Kuchinad et al., 2007). In contrast, chronic low back pain and complex regional pain syndrome are associated with bilateral hippocampal volume reduction, with hippocampus-amygdala gray matter loss closely linked to the severity of cognitive dysfunction in low back pain (Mutso et al., 2012; Zhou et al., 2022). Morphological changes in the prefrontal-thalamic circuit are observed not only in chronic tension-type headache patients (Apkarian et al., 2004; Schmidt-Wilcke et al., 2005) but also in individuals with neuropathic and non-neuropathic chronic back pain, underscoring the circuit’s central role in the pain–cognition interaction (Apkarian et al., 2004). Different pain types exhibit distinct patterns of structural brain changes. Osteoarthritis and fibromyalgia predominantly affect the PFC and hippocampus (Kuchinad et al., 2007; Murata et al., 2017), whereas chronic low back pain and phantom limb pain are characterized by gray matter reductions in the thalamus and neocortex (Apkarian et al., 2004; Ng et al., 2018). These structural abnormalities likely disrupt default mode network function, impairing memory encoding and information integration. In patients with mild cognitive impairment (MCI), bilateral amygdala–hippocampal atrophy serves as a core imaging marker and demonstrates accelerated hippocampal volume loss relative to non-MCI populations (Driscoll et al., 2009; Nickl-Jockschat et al., 2012). Systematic reviews further confirm that approximately 75% of studies on chronic low back pain report widespread gray matter volume reductions across multiple brain regions, with thalamic and neocortical changes exacerbating functional impairments by disrupting sensory–cognitive information processing (Ng et al., 2018; Zhou et al., 2022). Collectively, neuroimaging evidence supports a mechanistic link between chronic pain and cognitive dysfunction via gray matter remodeling across diverse brain regions. However, heterogeneity among studies and a lack of longitudinal data limit clinical translation. Therefore, integrating multimodal imaging with molecular biomarkers to characterize the dynamic evolution of brain plasticity is essential for advancing mechanistic understanding and informing intervention strategies.

## 3 Cognitive dysfunction associated with chronic pain: basic research

### 3.1 Basic research

The clinical evidence summarized in Table 1 indicates that chronic pain–related cognitive dysfunction most consistently affects attention, working memory, and episodic memory, with broader multi-domain impairments observed in fibromyalgia compared to more selective deficits, such as attention or processing speed decline, in osteoarthritis and chronic low back pain. These domain-specific patterns are echoed in the preclinical data presented in Table 2, where neuropathic pain models predominantly reproduce impairments in working memory, spatial learning, and recognition memory; deficits largely attributable to hippocampal and prefrontal cortex dysfunction. The convergence of mechanisms between clinical and preclinical studies, particularly synaptic plasticity impairment, neuroinflammation, and neurotransmitter dysregulation, reinforces the translational validity of these models and suggests that therapeutic strategies should prioritize restoring hippocampal–cortical network integrity and modulating neuroimmune activity.

**Table 2:** Cognitive function studies in mouse models of neuropathic pain.

Pain model	Animal species	Behavioral paradigm	Cognitive domain	Brain region	Key findings	Reference
CCI	Adult male mice	MWM, FCT, Y-maze, OFT	Learning and memory	Hip, mPFC, ACC	1. CCI mice exhibited persistent pain and cognitive impairment from postoperative days 21–28. 2. CCI-induced CHOP upregulation impaired synaptic plasticity and neuronal activity, contributing to chronic pain-associated cognitive deficits.	(Du et al., 2021; Zhang G-F, et al., 2021; Liu et al., 2022; Meng et al., 2025; Jiang et al., 2024)
SNL	Male Sprague–Dawley rats	Morris Water Maze	Spatial learning, Memory retention	Hip	1. SNL rats showed impaired cognitive function, which was significantly ameliorated by exendin-4 treatment. 2. SNL-induced chronic pain activated microglia and astrocytes in the hippocampal dentate gyrus, triggering neuroinflammatory cascades.	(Cui et al., 2020)
CFA	APP/PS1 transgenic mice	Morris Water Maze	Spatial learning, Memory function	Hippocampal CA1 and CA3 regions	1. Chronic pain accelerated the onset of spatial learning and memory deficits. 2. Neurotoxicity from chronic pain-induced NMDAR subunit dysregulation directly contributed to cognitive impairment.	(Gong et al., 2017)
SNI	Adult male Sprague–Dawley rats	NOR, Y-maze	Recognition memory	Hip, mPFC	1. SNI rats displayed reduced recognition indices at 14 days post-injury, indicating impaired cognitive function. 2. Increased hippocampal acetylated α-tubulin levels suppressed synaptic plasticity, exacerbating cognitive deficits.	(Palazzo et al., 2016; You et al., 2018)
PSNL	Male ddy mice	NOR	Learning, Recognition memory	Hip	1. PSNL mice exhibited significant cognitive impairment. 2. Reduced dendritic length and complexity in the hippocampus correlated with neuronal degeneration.	(Hisaoka-Nakashima et al., 2022b; Hisaoka-Nakashima et al., 2022a)
SNI	Male C57BL/6 mice	Morris Water Maze, OFT	Spatial learning and memory	PFC	1. SNI-induced neuropathic pain impaired spatial memory in middle-aged mice. 2. Gut microbiota significantly influenced cognitive function and pain modulation.	(Hua et al., 2022)
CFA	Wild-type and knockout mice	FCT	Learning and memory function	Cerebral cortex, Hip	1. Mice exhibited hippocampal-independent cognitive deficits. 2. Elevated IL-6 levels and reduced PSD-95 expression in the cerebral cortex contributed to cognitive impairment.	(Yang et al., 2014)
SNI	9-week-old C57BL6 Narp−/− transgenic mice	NOR, FCT	Learning and memory	Hip; Cortex	1. SNI impaired cognitive function in mice. 2. Downregulated NPTX2 expression in the hippocampus and cortex contributed to deficits.	(Wang et al., 2021)
SNI	3-month-old male mice	Y-maze, NOR	Working memory, Long-term memory	Hip	1. SNI impaired working memory and reduced long-term memory. 2. Hippocampal plasticity alterations drove cognitive deficits.	(Tyrtyshnaia et al., 2021)
SNI	8-week-old male C57BL/6 J mice	Y-maze; NOR	Spatial memory, Learning, Long-term memory	PFC; Hip	1. Memory deficits emerged at 1 month post-SNI but normalized by 12 months. 2. Impaired LTP in the prefrontal cortex-NAc core pathway and upregulated inflammation/apoptosis-related genes were observed.	(Guida et al., 2022)
SNI	Adult male Sprague–Dawley rats, C57 mice	Eight-Arm Radial Maze Test	Working memory, Short-term memory	Hip	1. SNI impaired spatial working memory and short-term memory in rodents. 2. TNF-α elevation disrupted hippocampal structure and function, inducing memory deficits.	(Ren et al., 2011)
PSNL	7-week-old male C57BL/6 J mice	Y-maze, NOR	Working memory, Recognition memory	PFC	1. PSNL induced working and recognition memory deficits at 6 months post-surgery. 2. Reduced global DNA methylation and downregulated methylation-related genes in the PFC drove cognitive impairment.	(Jang et al., 2021)
SNT	Male and female C57BL/6 J mice	MWM, NOR, OLR	Spatial learning and memory, Cognitive deficits	NA	SNT-induced neuropathic pain caused cognitive deficits in male mice but not females.	(You et al., 2018)
CCI	7–8-week-old male C57BL/6 J mice	Y-maze, NOR, OFT	Spatial working memory, Recognition memory	mPFC, Hip	1. CCI impaired memory function. 2. Hippocampal myelin loss and reduced neuronal activity were observed post-CCI.	(Zheng et al., 2023; Zhu et al., 2024)
CCI	3-month-old male C57Bl/6 mice	Y-maze, Passive Avoidance Test	Working memory, Long-term memory	Hip	1. Long-term memory impairment and working memory decline were observed. 2. Hippocampal neuroplasticity changes correlated with deficits.。	(Tyrtyshnaia and Manzhulo, 2020)
SNL	Male Sprague Dawley rats	NOR	Short-term and long-term memory	NA	SNL animals exhibited memory deficits only under high task difficulty.	(Phelps et al., 2021b; Phelps et al., 2021a)
SNI	8-week-old Sprague Dawley rats	Y-maze, NOR	Working memory, Learning and memory function	Hip	1. Cognitive impairment emerged at 22–24 days post-SNI. 2. CB2 receptors modulated microglial morphology/function via the DUSP6/ERK/NF-κB pathway.	(Xu et al., 2024)
SNI	Male C57BL/6 J APP/PS1 mice	MWM	Spatial learning and memory	Hip	1. APP/PS1 mice developed severe spatial learning/memory deficits post-SNI. 2. CCL2/CCR2 signaling suppressed hippocampal neurogenesis, exacerbating cognitive impairment.	(Chen J. et al., 2023)
CCI	TLR3 KO mice, C57BL/6 WT male mice	OFT, Y-maze, NOR, MWM	Working memory, Recognition memory, Spatial learning and memory	Hip	1. CCI induced cognitive decline. 2. TLR3 activation triggered neuroinflammation, apoptosis, and synaptic plasticity deficits.	(Zhang X. et al., 2023)
SNI	Adult male Sprague–Dawley rats (8–10 weeks)	Y-maze, NOR, OFT	Spatial memory, Working memory	Hip	1. SNI rats showed significant cognitive dysfunction. 2. Increased GABAARs-α5 expression attenuated inhibitory synaptic transmission, exacerbating deficits.	(Xiong et al., 2020)
SNI	Male C57 BL/6 J (8 weeks)	FCT, OFT, NOR, OLT	Learning and memory function	Hip	1. SNI induced cognitive dysfunction. 2. Hippocampal neuroinflammation, microglial M1 polarization, and synaptic loss were observed.	(Han et al., 2024c; Liu et al., 2024)
PSNL	Male ddy mice (5 weeks)	NOR, Y-maze	Recognition memory, Spatial memory	Hip	1. PSNL mice showed cognitive impairment at 2–4 weeks post-surgery. 2. Hippocampal microglial activation and neuroplasticity changes drove deficits.	(Hisaoka-Nakashima et al., 2022b; Hisaoka-Nakashima et al., 2022a)

Hip, Hippocampal; NMDA, N-methyl-D-aspartic acid receptor (R) subunits; FMT, Fecal microbiota transplantation; eCB, endocannabinoid system; OFT, Open field test; OLR, Object location recognition; APP/PS1, amyloid precursor protein/presenilin 1; OLT, Object location test; CPP, Conditioned Place Preference; LTP, long-term potentiation; HDAC, Histone Deacetylase.

#### 3.1.1 Cognitive impairments in chronic pain models

Across neuropathic pain models, hippocampal dysfunction emerges as the most consistent neuropathological feature, with structural alterations such as reduced dendritic complexity and spine density, as well as synaptic plasticity impairments including deficits in long-term potentiation (LTP; Wang et al., 2021; Hisaoka-Nakashima et al., 2022b; Hisaoka-Nakashima et al., 2022a; Jiang et al., 2024). Neuroinflammation is another recurring feature, characterized by microglial and astrocytic activation, elevated proinflammatory cytokines (e.g., IL-6, TNF-*α*), and subsequent neuronal apoptosis (Palazzo et al., 2016; Cui et al., 2020). Epigenetic modifications, such as histone deacetylase overexpression and global DNA hypomethylation, along with neurotransmitter receptor changes (e.g., NMDA receptor subunit imbalance, GABAAR-α5 upregulation), further contribute to cognitive deficits (Jang et al., 2021; Cai et al., 2022). Notably, chronic constriction injury (CCI) and spared nerve injury (SNI) models in APP/PS1 transgenic mice replicate both pain-induced memory impairment and amyloid pathology (Gong et al., 2017; Chen L. et al., 2023), offering unique value for studying the comorbidity of chronic pain and neurodegenerative disease.

Common rodent models, including CCI, SNI, spinal nerve ligation (SNL), partial sciatic nerve ligation (PSNL), and complete Freund’s adjuvant (CFA) induction—have consistently demonstrated significant cognitive impairment following neuropathic pain (Palazzo et al., 2016; Wang et al., 2021; Hisaoka-Nakashima et al., 2022b; Hisaoka-Nakashima et al., 2022a). Most studies have focused on spatial learning, memory, and attention. In Morris water maze (MWM) testing, SNI, SNL, and CCI animals exhibit clear spatial learning and memory deficits (Du et al., 2021; Hua et al., 2022; Chen L. et al., 2023). CCI mice show persistent pain and cognitive decline 21–28 days post-surgery in both MWM and fear conditioning tests (FCT; Zhang Y. et al., 2023), along with reduced spontaneous alternation rates in Y-maze and lower novel object recognition (NOR) performance at 14–21 days (Zheng et al., 2023; Zhu et al., 2024). Similarly, SNI rats spend less time exploring novel objects in NOR tests, and SNI mice show reduced alternation behavior and impaired object recognition within 1 month, with partial recovery after 12 months (Guida et al., 2022; Liu et al., 2024). PSNL models induce progressive deficits from 2 weeks to 6 months, affecting both alternation rates and NOR indices (Jang et al., 2021; Hisaoka-Nakashima et al., 2022b; Hisaoka-Nakashima et al., 2022a). Long-term, working, and short-term memory impairments are frequent across species (Phelps et al., 2021b; Phelps et al., 2021a; Cai et al., 2022; Xu et al., 2024), though some studies report no detectable changes within the first week (Zhang X. et al., 2023), suggesting that cognitive impairment is closely linked to the chronicity of nociceptive processing.

#### 3.1.2 Sex differences and hormonal regulation

Sex hormones exert profound modulatory effects on both nociception and cognition, providing a plausible mechanistic basis for the sex differences observed in chronic pain–related cognitive deficits. Estrogen, in particular, enhances hippocampal-dependent learning and memory by promoting dendritic spine formation, facilitating long-term potentiation (LTP), and modulating glutamatergic and cholinergic signaling (Ebner et al., 2015; Hara et al., 2015). It also exerts potent anti-inflammatory effects in the central nervous system (CNS), attenuating microglial activation and downregulating proinflammatory cytokines such as TNF-α and IL-1β (Liu et al., 2017; Fiore and Austin, 2018). These actions may protect female animals from hippocampal and prefrontal cortical dysfunction during chronic pain states. Progesterone similarly supports cognitive resilience by promoting myelin repair, enhancing GABAergic inhibition, and regulating neurosteroid synthesis, thereby reducing excitotoxicity (Kummer et al., 2020).

In contrast, testosterone has been shown to influence both pain sensitivity and cognitive performance in males, with declining levels associated with increased neuroinflammation, impaired synaptic plasticity, and deficits in spatial memory (Shansky et al., 2010; Han et al., 2024b). Androgen receptors in the hippocampus and prefrontal cortex regulate gene expression related to neurogenesis, axonal growth, and dopaminergic signaling, which may underlie the male-specific vulnerability to chronic pain–induced memory impairment (Cardoso-Cruz et al., 2019a). Moreover, fluctuations in sex hormone levels, such as those occurring across the estrous cycle, menopause, or andropause, can dynamically alter the neural substrates of pain and cognition, contributing to temporal variability in symptom severity (Cardoso-Cruz et al., 2022).

At the molecular level, sex hormones modulate epigenetic landscapes in pain- and cognition-related brain regions. Estrogen receptor activation can induce histone acetylation at promoters of synaptic plasticity genes, while testosterone depletion has been linked to increased DNA methylation of genes involved in neurotrophic signaling (Journée et al., 2023). These epigenetic effects may partly explain the persistence or reversibility of cognitive deficits in chronic pain conditions. Taken together, hormonal modulation represents a critical axis for understanding sex-specific cognitive outcomes in chronic pain, and future preclinical studies should incorporate hormone profiling and receptor-targeted interventions to better translate findings to clinical populations.

#### 3.1.3 Limitations of current models

Despite substantial progress, limitations remain. Different pain models yield heterogeneous cognitive outcomes, limiting cross-study comparability. Moreover, research has predominantly targeted spatial and working memory, with less emphasis on executive function, sustained attention, and other clinically relevant domains. Moving forward, comprehensive behavioral batteries and multimodal assessment strategies are essential to capture the full cognitive spectrum of chronic pain in preclinical settings and to bridge the translational gap between animal models and human pathology.

### 3.2 Involved potential mechanisms

**Figure 1:**
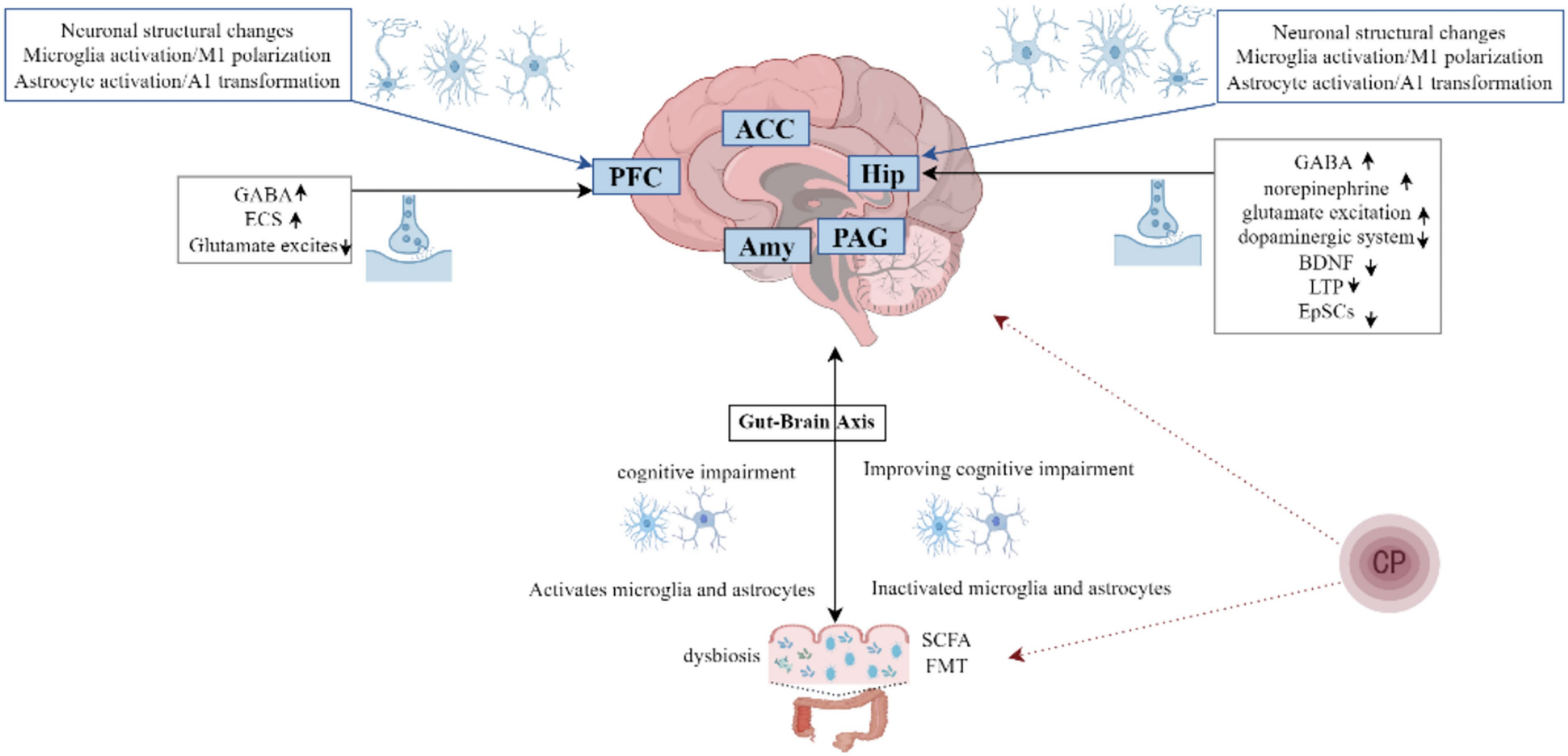
Potential mechanisms of cognitive dysfunction associated with chronic pain. Amy, Amygdala; PAG, Periaqueductal gray; SCFA, Short-chain fatty acid; FMT, Fecal microbiota transplantation; ECS, Endocannabinoid system; EpSCs, Excitatory postsynaptic currents; CP, Chronic pain.

In recent years, notable progress has been made in understanding the mechanisms underlying cognitive dysfunction in chronic pain, yet the processes by which chronic pain induces memory deficits remain complex. These impairments involve multiple brain regions, neural circuits, cell types, and molecular pathways, rather than being attributable to a single factor (Moriarty et al., 2011). Despite advances, current basic research remains limited in depth. Here, we integrate recent findings to summarize the pathological mechanisms contributing to chronic pain–related cognitive deficits (Figure 1).

#### 3.2.1 Functional brain regions and interactive mechanisms

Chronic pain may consume substantial cognitive resources, thereby reducing the capacity to perform complex cognitive tasks (Phelps et al., 2021b; Phelps et al., 2021a). Neurobiologically pain-related cognitive dysfunction is associated with structural and functional remodeling of distributed brain networks, with core pathological changes occurring in the hippocampus, PFC, and anterior cingulate cortex (ACC).

The hippocampus, critical for memory encoding and consolidation, exhibits impaired synaptic plasticity and heightened neuroinflammatory responses in neuropathic pain models. Reduced neurogenesis in the dentate gyrus (DG) is directly linked to short-term and recognition memory impairments (Kodama et al., 2011; Ren et al., 2011), alongside abnormalities in long-term potentiation (LTP; Kodama et al., 2007). Elevated hippocampal levels of pro-inflammatory cytokines such as TNF-*α*, IL-1β, and MCP-1 exacerbate neuroinflammation, further impairing cognition (Liu et al., 2017; Fiore and Austin, 2018). Following sciatic nerve injury, increased acetylated α-tubulin suggests that altered microtubule stability may disrupt synaptic plasticity and contribute to learning and memory deficits (You et al., 2018). Chronic inflammatory pain also induces selective hippocampal-independent memory deficits via neuroinflammation and synaptic loss (Yang et al., 2014). Rather than directly mediating nociception, the hippocampus may modulate pain-related behaviors indirectly through cognitive resource allocation (Xu et al., 2024).

The PFC, particularly the mPFC is central to executive functions, decision-making, and attention. Neural injury can inactivate the mPFC via glutamatergic synaptic inhibition, leading to decision-making deficits (Ji et al., 2010; Kummer et al., 2020). Disruption of the mPFC–dorsal hippocampus CA1 (mPFC–dCA1) circuit impairs memory (Han et al., 2024a), while optogenetic inhibition of glutamatergic neurons in the prelimbic cortex (PL)-mPFC pathway reverses neuropathic pain–related working memory deficits by restoring mPFC–dCA1 synchrony and local firing activity (Cardoso-Cruz et al., 2019b). The PL-mPFC exerts its influence partly through direct excitatory projections to the nucleus accumbens (NAcc) core and indirect modulation via interconnected neurons (Domingo-Rodriguez et al., 2020). Selective inhibition of PL-mPFC terminals projecting to the NAcc partially rescues spatial working memory deficits and modifies PFC–striatal connectivity without affecting nociceptive sensitivity (Cardoso-Cruz et al., 2022). These findings suggest that chronic pain disrupts PFC network integration, leading to impairments in recognition and spatial memory.

The ACC, a limbic structure integral to cognition, learning, memory, and decision-making, exhibits neuronal hyperactivity in chronic pain, characterized by increased spontaneous firing and an imbalance between excitatory and inhibitory signaling (Cardoso-Cruz et al., 2022). High-frequency stimulation of the ACC enhances pyramidal neuron activity, indicating that an excitatory/inhibitory (E/I) imbalance may reinforce maladaptive pain-related memory consolidation (Cardoso-Cruz et al., 2022; Zhu et al., 2022). Restoring oligodendrocyte myelination in the ACC can normalize network activity and alleviate cognitive impairments (Hasan et al., 2023). These findings underscore the need for detailed mapping of ACC subcircuits and their maladaptive plasticity during chronic pain.

Chronic pain–induced cognitive dysfunction arises from multi-regional interactions involving inflammatory mediator dysregulation (Wang et al., 2021), glutamate–GABA imbalance (Zhu et al., 2022), and synaptic plasticity abnormalities (Zhang X. et al., 2023). Persistent nociceptive input disrupts the E/I balance within the mPFC and ACC, impairing their normal processing capacity (Qi et al., 2022; Song et al., 2024). Amygdala-driven mPFC dysfunction plays a key role in pain-related cognitive impairments (Ji et al., 2010). Chronic visceral pain, for instance, disrupts theta oscillatory synchrony between the basolateral amygdala (BLA) and ACC, leading to executive deficits in visceral hypersensitive rats (Cao et al., 2016). Neuropathic pain reduces the excitability and synaptic efficiency of dorsal CA1 pyramidal neurons, decreasing glutamatergic input to the mPFC and thereby exacerbating pain sensitivity and cognitive decline (Han et al., 2024b). Additionally, the periaqueductal gray (PAG), a central hub for descending pain modulation, receives cortical inputs mainly from the anterior dorsal raphe (DR) and mPFC (Ong et al., 2019); dysfunction in the PAG–DR circuit may also contribute to cognitive impairments (Deng et al., 2023). These pain-induced behavioral changes are related to structural and functional alterations in multiple brain regions (May, 2008). Collectively, structural and functional alterations within the hippocampus, PFC, ACC, and interconnected regions form the neural substrate for chronic pain–associated cognitive dysfunction. The interplay between these regions, mediated by maladaptive neuroinflammation, disrupted synaptic signaling, and network-level dysregulation, offers multiple potential targets for therapeutic intervention.

#### 3.2.2 Molecular mechanisms of cognitive dysfunctions related to chronic pain

##### 3.2.2.1 Neurotransmitters and receptors

Cognitive dysfunction in chronic pain is closely associated with disruption of the excitatory–inhibitory (E/I) balance, involving GABAergic overactivation and glutamatergic hypofunction. GABA, the principal inhibitory neurotransmitter in the CNS, is abnormally elevated in the hippocampus and medial prefrontal cortex (mPFC) in neuropathic pain models, suppressing neural circuit activity (Medeiros et al., 2020; Tyrtyshnaia and Manzhulo, 2020). Neuropathic pain increases α5-subunit, containing GABAA*_A_* receptors (α5GABA*_A_* ARs) expression in parvalbumin- and somatostatin-positive interneurons, enhancing inhibitory drive, disrupting synaptic plasticity, and contributing to memory and learning deficits (Cai et al., 2022). In the mPFC, elevated GABA levels and reduced D-aspartate concentrations lead to network desynchronization, impairing working memory (Ji et al., 2010; Medeiros et al., 2020). Glutamatergic dysfunction is characterized by reduced N-methyl-D-aspartate receptor (NMDAR) activity and weakened excitatory synaptic transmission, impairing long-term potentiation (LTP) and hippocampal-dependent memory formation (Pal, 2021). Neuropathic pain models demonstrate decreased hippocampal glutamatergic transmission and LTP, highlighting the importance of glutamate signaling in neuroplasticity and pain-cognition interactions (Xiong et al., 2020).

Monoaminergic systems further modulate pain-related cognitive deficits. Norepinephrine (NE) in the hippocampus supports spatial memory through the locus coeruleus (LC)–hippocampal pathway, while aberrant NE signaling in the PFC is associated with attentional impairments (Suto et al., 2014; Mello-Carpes et al., 2016). Dopamine regulates hippocampal synaptic activity and spatial memory retention, with D_2_/D_3_ receptor expression influencing dorsal–ventral hippocampal connectivity (Cardoso-Cruz et al., 2014; Broussard et al., 2016). Serotonin (5-HT) alterations, including elevated hippocampal 5-HT, inhibit neurogenesis and contribute to cognitive decline (Song et al., 2016; Kędziora et al., 2023). Collectively, dysregulation of GABAergic, glutamatergic, and monoaminergic systems disrupts synaptic plasticity and network synchrony, underpinning chronic pain–associated cognitive dysfunction.

##### 3.2.2.2 Brain-derived neurotrophic factor

Chronic pain disrupts brain-derived neurotrophic factor (BDNF) signaling, contributing to cognitive dysfunction through multiple neural circuit and molecular mechanisms. In the BLA, excessive neuronal activation impairs PFC function via glutamatergic–GABAergic interactions, leading to decision-making deficits (Ji et al., 2010). In the APP/PS1 mouse model, chronic pain increases expression of the NR2B subunit of N-methyl-D-aspartate receptors (NMDARs) in the hippocampal CA3 region, shifting the NR2B/NR2A ratio toward neurotoxic signaling and thereby compromising synaptic plasticity and memory performance (Gong et al., 2017). In CCI models, elevated GABA and reduced glutamate and BDNF levels in the hippocampal CA1 region are associated with impairments in spatial learning and memory (Saffarpour et al., 2017). More broadly, neuropathic pain–induced reductions in hippocampal BDNF limit synaptic efficacy, whereas activation of the cAMP response element-binding protein (CREB)/BDNF pathway protects against pain-related cognitive decline (Zhang et al., 2022). Environmental enrichment in nerve-injured mice enhances long-term memory and synaptic plasticity through BDNF–tropomyosin receptor kinase B (TrkB) signaling (Wang et al., 2019). Similarly, stimulation of BDNF release from the ventral tegmental area (VTA) to the dentate gyrus (DG) restores hippocampal neurogenesis and reverses memory impairments (Xia et al., 2020). These findings indicate that BDNF serves as a critical mediator of synaptic plasticity and neurogenesis in chronic pain–associated cognitive impairment. Targeting the BDNF/TrkB pathway represents a promising therapeutic strategy, although the precise molecular mechanisms and circuit-specific actions warrant further elucidation.

##### 3.2.2.3 Endogenous cannabinoid system

The endogenous cannabinoid system (ECS) plays a key role in CNS development and may modulate pain–cognition interactions, thereby influencing the progression of neuropathic conditions in chronic pain (Hua et al., 2022). The ECS is composed of cannabinoid receptors (CBRs), endogenous ligands, and enzymes responsible for ligand synthesis and degradation. Two major receptor subtypes have been identified: CB1 receptors (CB1R) and CB2 receptors (CB2R). CB1R, predominantly expressed in the CNS, is critically involved in regulating pain perception, emotional processing, and cognitive functions (Chiou et al., 2013; Zou and Kumar, 2018; Karimi-Haghighi and Shaygan, 2025). Activation of CB1R enhances PFC output while suppressing amygdala activity, thereby attenuating pain-related emotional distress and reducing cognitive impairments (Karimi-Haghighi and Shaygan, 2025). CB2R, although less abundant in the CNS, exerts important modulatory effects on neuroinflammation; activation of hippocampal CB2R can reverse microglial dysfunction in chronic pain states (Xu et al., 2024). Restoration of endogenous cannabinoid signaling also influences glutamatergic modulation: activation of metabotropic glutamate receptor 5 (mGluR5) via ECS signaling increases limbic system output, which in turn suppresses pain behaviors (Kiritoshi et al., 2016). Through these multi-level mechanisms, the ECS coordinates neural activity between key regions such as the PFC, hippocampus, and amygdala, thereby regulating both nociceptive and cognitive processes.

Collectively, these findings highlight the ECS as a critical neuromodulatory network linking pain and cognition. Targeting CB1R and CB2R pathways, as well as downstream glutamatergic and neuroimmune signaling, represents a promising therapeutic approach for alleviating chronic pain–associated cognitive dysfunction.

##### 3.2.2.4 Gut-brain axis

The gut–brain axis represents a complex bidirectional communication network between the gastrointestinal tract and CNS, mediated through immune, neural, and endocrine pathways. Dysregulation of this axis has been implicated in the pathogenesis of chronic pain, neuroinflammation, and cognitive dysfunction via both peripheral and central mechanisms (Lin et al., 2020). Gut microbiota dysbiosis can disrupt intestinal barrier integrity, triggering systemic inflammation and contributing to pain hypersensitivity and cognitive impairments (Sampson and Mazmanian, 2015; Sun et al., 2019). Experimental evidence indicates that depletion of gut microbiota reduces oxidative stress and ameliorates mitochondrial dysfunction in microglia; however, prolonged antibiotic intervention can exacerbate microglial impairment. This occurs via decreased production of short-chain fatty acids (SCFAs), which promotes polarization toward the pro-inflammatory M1 phenotype and downregulates hippocampal synaptic protein expression, ultimately impairing spatial memory (Zhou F. et al., 2021; Magni et al., 2023). SCFAs, key microbial metabolites, can cross the blood–brain barrier and modulate neural function through epigenetic mechanisms. In chronic postoperative pain models, SCFAs improve histone acetylation and normalize synaptic transmission deficits in the mPFC, hippocampal CA1, and central amygdala (CeA) via the ACSS2–HDAC2 signaling axis, thereby mitigating pain-associated cognitive decline (Dalile et al., 2019; Li et al., 2022). Additionally, the gut–brain axis influences neuroinflammation and neurodegeneration by regulating astrocyte maturation and reactivity; the formation of reactive astrocytes represents a potential mechanism through which gut microbiota modulates neuropathological processes (Magni et al., 2023). Of note, interactions between gut microbiota and the endogenous cannabinoid (eCB) system—termed the microbiota–eCB axis—have emerged as critical modulators of both neuropathic pain and associated cognitive deficits (Hua et al., 2022).

Collectively, these findings suggest that the gut–brain axis contributes to the pathophysiology of chronic pain–related cognitive dysfunction through multiple mechanisms, including microbial metabolite signaling, immune modulation, epigenetic regulation, and neuroglial interactions. Furthermore, the gut–brain axis does not operate in isolation but interacts extensively with other pathophysiological mechanisms. For instance, microbiota-driven immune activation can amplify neuroinflammatory cascades, while SCFA-mediated epigenetic regulation may converge with synaptic plasticity alterations. Dysbiosis-induced astrocyte reactivity links directly to glial–neuronal interactions that exacerbate both pain hypersensitivity and cognitive decline. These multidirectional connections underscore the integrative nature of chronic pain–related cognitive dysfunction and support the need for schematic representation linking the gut–brain axis, neuroinflammation, and cognitive deficits, thereby providing a more cohesive framework for understanding and targeting this complex pathology. Targeting gut microbiota composition and function may thus represent a promising therapeutic approach.

##### 3.2.2.5 Translational limitations and clinical significance

Despite extensive mechanistic insights from preclinical studies, translating these findings into effective clinical interventions for chronic pain–related cognitive dysfunction remains challenging. Most evidence originates from rodent models (e.g., SNI, CCI, APP/PS1), which cannot fully capture the complexity, heterogeneity, and chronicity of human pain conditions. In addition, animal studies rarely incorporate common comorbidities such as depression, anxiety, sleep disturbances, or metabolic disorders, and often do not reflect demographic variability including age, sex, and genetic background. Methodological differences further complicate translation: cognitive performance in animals is typically assessed via maze navigation, fear conditioning, or operant tasks, whereas clinical studies rely on standardized neuropsychological tests targeting specific domains. Species-specific differences in pharmacokinetics, drug metabolism, and dosing regimens also contribute to discrepancies in therapeutic efficacy.

Nevertheless, elucidating the molecular and circuit-level mechanisms underlying pain-associated cognitive deficits holds substantial clinical significance. Identification of key pathways may guide biomarker development, predict cognitive vulnerability, and inform individualized therapeutic strategies. Integrative translational approaches, such as human neuroimaging, neuropsychological assessment, multi-omics profiling, and gut microbiota analysis, are essential to validate preclinical findings and bridge mechanistic insights to clinical application. Such strategies can support the design of targeted interventions, preventive measures, optimized pharmacological treatments, and personalized cognitive rehabilitation protocols, ultimately advancing precision medicine in chronic pain management.

#### 3.2.3 Cellular mechanisms of cognitive dysfunctions related to chronic pain

Cellular damage within the CNS constitutes a key pathological substrate underlying chronic pain–associated cognitive dysfunction. Pain-related activation of neurons and glial cells in the PFC and hippocampus promotes abnormal release of pro-inflammatory mediators, disrupts synaptic plasticity, and contributes to behavioral deficits (Mohammadi et al., 2020; Yao et al., 2024). Central to this process is the phenotypic transformation of glial cells, particularly the polarization of microglia toward the pro-inflammatory M1 phenotype and the transformation of astrocytes into the neurotoxic A1 subtype. These changes disrupt neuron–glia homeostasis and exacerbate cognitive decline.

##### 3.2.3.1 Neuroglial cells

Microglia, the resident immune cells of the CNS, exert a bidirectional influence on pain-related cognitive dysfunction through dynamic regulation of M1/M2 phenotypic states. Under physiological conditions, microglia support cognitive function via synaptic pruning and neurotransmitter homeostasis. Pathological activation drives M1 polarization, leading to the release of pro-inflammatory cytokines such as TNF-*α* and IL-1β, which amplify neuroinflammation and impair memory (Saffarpour et al., 2021; Han et al., 2024a). In the SNI model, hippocampal M1 polarization correlates strongly with cognitive deficits. Activation of liver X receptors (LXRs) suppresses the M1 phenotype via the PI3K/AKT pathway, thereby attenuating neuroinflammation and restoring synaptic plasticity (Han et al., 2022).

Microglial–astrocytic co-activation in the dentate gyrus (DG) further amplifies the neuroinflammatory cascade, as demonstrated in the SNL model (Cui et al., 2020). Overexpression of IL-1β in the hippocampus severely impairs both contextual and spatial memory (Hein et al., 2010), while excessive TNF-α release induces passive avoidance deficits, inhibits long-term potentiation (LTP), and disrupts hippocampal synaptic plasticity (Butler et al., 2004; Ren et al., 2011). Similarly, IL-6 overproduction reduces LTP and triggers widespread memory impairments (Tancredi et al., 2000). Inhibition of microglial activation or blockade of high-mobility group box 1 (HMGB1) release can prevent chronic pain–induced cognitive decline (Hisaoka-Nakashima et al., 2022b; Hisaoka-Nakashima et al., 2022a). Although complete microglial depletion can reverse memory deficits (Ren et al., 2011; Liu et al., 2017), therapeutic strategies that promote a shift toward the neuroprotective M2 phenotype appear more promising (Wang et al., 2022).

Astrocytes also undergo pathological remodeling during chronic pain. In the PFC and hippocampus, astrocytes initially exhibit reactive hyperplasia (Cui et al., 2020; Asgharpour-Masouleh et al., 2023), but later progress to numerical reduction and atrophy due to sustained neurotoxicity (Zhang Y. et al., 2023), a stage-dependent transformation potentially linked to pain duration. Functionally, aberrant astrocytic lactate metabolism reduces excitability of hippocampal CA1 pyramidal neurons, impairing spatial memory (Han et al., 2024). Moreover, downregulation of aquaporin-4 (AQP4) disrupts glymphatic clearance, accelerating neurodegeneration (Zhang Y. et al., 2023). These findings underscore glial-mediated neuroinflammation as a key target for mitigating pain-associated cognitive impairments.

##### 3.2.3.2 Neurons

Chronic pain disrupts cognitive processes through multifaceted impairments in hippocampal and PFC neuronal function, particularly by altering synaptic plasticity. These changes involve dysregulation of synaptic protein expression, dendritic morphology, and intracellular signaling pathways (Hisaoka-Nakashima et al., 2022b; Hisaoka-Nakashima et al., 2022a; Meng et al., 2025). In neuropathic models, hippocampal synaptic plasticity deficits contribute directly to memory impairment (Mutso et al., 2012). Chronic pain reduces postsynaptic density protein expression, diminishes glutamatergic transmission, as evidenced by reduced NMDA/AMPA currents and impaired excitatory postsynaptic currents, and selectively impairs LTP without significantly affecting long-term depression (LTD; Kodama et al., 2007; Xiong et al., 2020). The precise contribution of LTP/LTD imbalance to neural circuit dysfunction remains to be clarified.

Changes in synaptic plasticity, encompassing functional and structural plasticity, are pivotal in memory formation (Yang et al., 2009). Structurally, chronic pain reduces dendritic spine density, dendritic complexity, axonal branching, and hippocampal neurogenesis (Guida et al., 2022; Hisaoka-Nakashima et al., 2022b; Hisaoka-Nakashima et al., 2022a). In the SNI model, hippocampal neurons display shortened dendrites and reduced AMPA receptor expression, correlating with spatial memory decline (Tyrtyshnaia and Manzhulo, 2020). CCI similarly decreases dendritic spine density and synapse-related protein levels, paralleling deficits in memory performance (Tang et al., 2024). Reduced excitatory synapse numbers and impaired neuronal plasticity further compromise network information integration (Xiong et al., 2020). Neuroinflammation is a major driver of these neuronal alterations. Persistent hippocampal inflammation inhibits LTP formation, accelerates dendritic atrophy, and promotes myelin loss through glial-derived inflammatory mediators (Lecca et al., 2022; Zhu et al., 2024). Across multiple animal models, sustained glial activation and cytokine overproduction converge on the inhibition of neurogenesis and synaptic remodeling (Mai et al., 2021). In summary, chronic pain impairs cognition through a complex interplay of neuroinflammatory processes and structural–functional synaptic deficits, disrupting the dynamic balance essential for memory and learning.

## 4 Interventional treatments

The clinical management of chronic pain traditionally relies on pharmacological agents such as opioids, non-steroidal anti-inflammatory drugs, and neuromodulators. While these medications can achieve effective analgesia, they are often accompanied by adverse effects, including an increased risk of cognitive impairment. Owing to the limitations of conventional drug therapies, current research efforts are increasingly focused on the development of targeted pharmacological agents and the refinement of non-pharmacological strategies. The overarching goal is to preserve analgesic efficacy while minimizing cognitive side effects, thereby improving the safety and effectiveness of long-term chronic pain management.

### 4.1 Clinical interventional treatments

#### 4.1.1 Pharmacological interventions

Commonly prescribed analgesics for chronic pain exhibit bidirectional effects on cognitive function. Agents such as gabapentin, opioids, and N-methyl-D-aspartate receptor (NMDAR) antagonists have been reported to impair domains including memory, executive function, and attention (Shem et al., 2018; Pask et al., 2020). The cognitive impact of opioids remains controversial: while some studies indicate potential cognitive improvement in patients with conditions such as low back pain (Jamison et al., 2003; Tassain et al., 2003), the preponderance of evidence associates chronic opioid therapy with measurable cognitive deficits in this population (Kurita et al., 2015; Richards et al., 2018). Specifically, morphine administration has been shown to induce transient anterograde and retrograde memory impairments (Kamboj et al., 2005), although no consistent correlation has been established between cognitive decline and opioid dosage or treatment duration (Sjøgren et al., 2005). Similarly, repeated exposure to NMDAR antagonists such as ketamine can lead to spatial memory deficits, likely linked to reduced activation of the hippocampus and parahippocampal gyrus (Morgan et al., 2014). While short-term analgesia or relief from pain-related stress may indirectly enhance cognitive performance (Wolrich et al., 2014; Ferreira et al., 2016), prolonged use of these agents tends to exacerbate cognitive impairment risk. To address this issue, future investigations should systematically characterize the dose–response relationship by considering the type of medication, its dosage, and treatment duration. Such analyses are essential to clarify the causal association between analgesic therapy and cognitive performance in chronic pain patients.

#### 4.1.2 Non-pharmacological treatments

##### 4.1.2.1 Cognitive behavioral therapy

Cognitive Behavioral Therapy (CBT) is among the most extensively validated psychological interventions for chronic pain management. It ameliorates pain-related cognitive dysfunctions via multidimensional mechanisms. Evidence indicates that CBT attenuates the stress response in chronic pain patients by modulating hypothalamic–pituitary–adrenal axis activity, thereby mitigating the detrimental cognitive effects of neuroendocrine dysregulation (Eller-Smith et al., 2018). Neuroimaging studies further demonstrate that CBT can reverse gray matter volume loss in the PFC and sensory cortices of chronic pain patients, promoting the normalization of aberrant neural activity patterns (Yoshino et al., 2018). In older populations, combining CBT with structured physical exercise has been shown to significantly reduce pain intensity, improve functional capacity, and attenuate pain-related maladaptive cognition, although the benefits are primarily observed in pain-related rather than generalized cognitive outcomes (Cheng et al., 2022). Randomized controlled trials have further confirmed that integrated CBT protocols effectively reduce pain catastrophizing, enhance daily activity performance, and improve overall health status (Lackner et al., 2024; Lee et al., 2024). When implemented in conjunction with other therapeutic modalities, CBT may enhance patient outcomes by addressing both psychological and neurobiological contributors to pain-related cognitive impairment.

##### 4.1.2.2 Transcranial magnetic stimulation and transcranial direct current stimulation

Transcranial Magnetic Stimulation (TMS) and Transcranial Direct Current Stimulation (tDCS) are non-invasive neuromodulatory approaches that target the prefrontal–hippocampal circuitry, representing promising strategies for the management of chronic pain–associated cognitive deficits. Repetitive TMS (rTMS) can reorganize dysfunctional neural networks and exert anti-neuroinflammatory effects, while tDCS modulates cortical excitability with polarity-specific effects—anodal stimulation lowers neuronal firing thresholds, and cathodal stimulation raises them (Bai et al., 2023; Moshfeghinia et al., 2023). Clinical evidence suggests that anodal tDCS applied to the dorsolateral prefrontal cortex (DLPFC) can enhance orienting and executive attention in patients with fibromyalgia, potentially through long-term potentiation (LTP) induction (Silva et al., 2017). In healthy individuals, tDCS has demonstrated efficacy in improving attention, learning, memory, and working memory (Coffman et al., 2014; Carvalho et al., 2015). Transcranial random noise stimulation (tRNS), which delivers stochastic alternating current patterns to induce resonance-based neuronal synchronization, has been shown to both alleviate fibromyalgia symptoms and enhance working memory. Compared to conventional tDCS, tRNS exhibits broader and more sustained effects (Curatolo et al., 2017). Target selection is critical: DLPFC stimulation can concurrently ameliorate anxiety, depression, and cognitive impairments, whereas primary motor cortex stimulation primarily yields analgesic benefits (Curatolo et al., 2017), From a therapeutic perspective, CBT promotes psychological and cognitive reorganization through top-down mechanisms, while TMS and tDCS facilitate bottom-up modulation of synaptic plasticity and network connectivity. Together, these complementary approaches provide a framework for precision multimodal interventions targeting both psychological and neurophysiological domains of chronic pain–related cognitive dysfunction. A summary of clinical intervention treatments is provided in Table 3.

**Table 3:** Clinical interventions for cognitive dysfunction associated with chronic pain.

Intervention	Age (years)	Pain type	Cognitive outcomes	Reference
Gabapentin	19–59	Spinal cord injury	Significant decline in memory, executive function, and attention	(Shem et al., 2018)
Morphine	65.2 ± 12.2	Chronic low back pain and cancer pain	Memory impairment	(Kamboj et al., 2005)
tDCS	18 ~ 65	Fibromyalgia	Enhanced directed and executive attention performance	(Silva et al., 2017)
Exercise and cognitive behavioral therapy	≥60	Multisite chronic pain	Improved cognitive performance in chronic pain patients	(Cheng et al., 2022)
tRNS	26 ~ 67	Fibromyalgia	Effective alleviation of cognitive deficits	(Andrews et al., 2011)
CBT	≥18	chronic pain	Potential improvement in pain catastrophizing	(Taguchi et al., 2021; Lee et al., 2024)

### 4.2 Preclinical therapeutic interventions

#### 4.2.1 Pharmacological interventions

Emerging therapeutic strategies for chronic pain-associated cognitive dysfunction increasingly emphasize multi-target approaches. Pharmacological interventions targeting neuroinflammation and neurotrophic regulation have shown promising preclinical efficacy. For instance, infliximab can reverse neuroinflammation and restore hippocampal neurogenesis, thereby improving cognitive function (Yao et al., 2024). Curcumin and its nanoformulations attenuate neuropathic pain and memory deficits by reducing hippocampal IL-1β and TNF-*α* levels and repairing synaptic ultrastructure (Zhang et al., 2018; Du et al., 2021). Similarly, flurbiprofen ester and oral magnesium levetiracetam inhibit neuroinflammatory responses, alleviating both neuropathic pain and associated cognitive impairments (Zhou X. et al., 2021; Huang et al., 2022). Modulation of the glutamatergic system represents another therapeutic avenue. The NMDA receptor agonist d-aspartate restores glutamate transmission and improves cognitive deficits (Palazzo et al., 2016), whereas the NMDA receptor antagonist memantine protects spatial memory by preventing postoperative hippocampal LTP impairment (Morel et al., 2013). Additionally, chloramphenicol promotes myelin regeneration, mitigating CCI-induced reductions in neuronal activity and enhancing memory function (Zhu et al., 2024). Synaptamide has also been shown to reverse dendritic spine loss and restore LTP, thereby improving working memory (Tyrtyshnaia et al., 2021). Epigenetic regulation offers further potential. The methyl donor S-adenosylmethionine (SAM) preserves DNA methylation in the prefrontal cortex, alleviating cognitive decline (Grégoire et al., 2017), while SCFAs enhance synaptic transmission through histone acetylation. Preclinical evidence also supports the neuroprotective effects of anti-TNF-α, anti-IL-1β, anti-IL-6 agents, and endocannabinoid-like compounds (Lowe et al., 2021; Ortí-Casañ et al., 2022). Despite these advances, clinical translation remains challenging due to limitations in pharmacodynamic stability, blood–brain barrier permeability, and long-term safety. Future research should integrate multi-omics approaches with cross-scale neuroimaging to develop combination therapies that simultaneously target neuroinflammation, synaptic plasticity, and epigenetic modulation, ultimately achieving both pain alleviation and cognitive protection.

#### 4.2.2 Non-pharmacological interventions

Acupuncture has emerged as a promising non-pharmacological strategy for alleviating chronic pain and associated cognitive deficits following peripheral nerve injury, demonstrating multi-target regulatory potential. Preclinical studies indicate that acupuncture can restore epigenetic homeostasis by modulating DNA methylation in the PFC. Specifically, it reverses chronic pain-induced methylation abnormalities of genes such as *Nr4a1* and *Rasgrp1*, and normalizes global DNA methylation patterns in the PFC, periaqueductal gray, hippocampus, and amygdala (Jang et al., 2021). Electroacupuncture has been shown to enhance cognitive function via synergistic mechanisms. Four weeks of continuous treatment significantly increase mechanical pain thresholds, and hippocampal proteomic analyses have identified molecular correlates underlying improvements in neuropathic pain-associated cognitive deficits (Jang et al., 2019; Gong et al., 2021).

Collectively, these findings suggest that acupuncture can modulate the “pain–neuroinflammation–cognitive impairment” axis through dual mechanisms: epigenetic regulation and synaptic functional remodeling. This evidence highlights acupuncture as a novel, multi-target, non-pharmacological intervention for managing chronic pain comorbidities. A summary of preclinical research on therapeutic interventions is provided in Table 4.

**Table 4:** Preclinical interventions for cognitive dysfunction in chronic pain models.

Intervention	Animal species	Pain type	Cognitive impact	Mechanism of action	Reference
Memantine	Adult male Sprague–Dawley rats	Chronic neuropathic pain	Alleviates spatial memory deficits	NMDAR antagonism	(Morel et al., 2013)
Flurbiprofen axetil	Adult Sprague–Dawley rats	Inflammatory pain	Improves mild cognitive impairment	Reduces hippocampal neuronal damage and pro-inflammatory cytokine release	(Huang et al., 2022)
Electroacupuncture	Male Sprague–Dawley rats	Chronic neuropathic pain	Eliminates memory deficits	Modulates hippocampal inflammatory protein levels, suppresses microglial M1 polarization, and reduces neuroinflammation	(Gong et al., 2021)
MR16-1	Male ddy mice	Neuropathic pain	Improves cognitive impairment	Prevents dendritic complexity loss and neuronal degeneration in the hippocampus	(Hisaoka-Nakashima et al., 2022b; Hisaoka-Nakashima et al., 2022a)
Curcumin	Adult male Sprague Dawley rats	Trigeminal neuropathic pain	Improves spatial learning and memory deficits	Repairs hippocampal neuronal and synaptic damage	(Zhang et al., 2018)
d-Asp	Male 5-week-old CD1 mice	Neuropathic pain	Reduces cognitive impairment	Restores amino acid release in the mPFC and rescues postsynaptic protein expression	(Palazzo et al., 2016)
(S)-ketamine	Adult male C57BL/6 J mice	Chronic neuropathic pain	Ameliorates spatial working memory deficits	Downregulates hippocampal HDAC2, upregulates BDNF levels, and partially normalizes gut microbiota composition	(Jiang et al., 2024)
Infliximab	Adult male Sprague–Dawley rats	Neuropathic pain	Attenuates spatial memory impairment	Inhibits hippocampal astrocyte/microglial activation, reduces pro-inflammatory cytokines and restores dentate gyrus neurogenesis	(Yao et al., 2024)
Synaptamide	3-month-old male mice	Neuropathic pain	Improves working and long-term memory	Reverses dendritic spine density loss and suppresses microglial activation	(Tyrtyshnaia et al., 2021)
Acupuncture	7-week-old male C57BL/6 J mice	Neuropathic pain	Alleviates cognitive dysfunction	Modulates DNA methylation, mitochondrial dysfunction-related genes, and enhances hippocampal NR2B/GluR1 expression and synaptic plasticity	(Jang et al., 2019, 2021)
Metformin	C57BL/6 J wild-type mice	Neuropathic pain	Reverses pain-induced cognitive deficits	Restores infralimbic parvalbumin loss	(Shiers et al., 2018)
SAM	Male CD1 mice	Neuropathic pain	Reverses cognitive impairment	Restores global DNA methylation in the frontal cortex	(Grégoire et al., 2017)
Resveratrol	Adult male Sprague–Dawley rats	Trigeminal neuralgia	Improves learning and memory deficits	Restores hippocampal ultrastructure and activates the CREB/BDNF pathway	(Saffarpour et al., 2017)
Cannabidiol	Male Wistar rats	Neuropathic pain	Enhances cognitive performance	Induces neuroplasticity via recruitment of the CA1-PrL pathway	(Medeiros et al., 2024)
Duloxetine	Male Sprague Dawley rats	Neuropathic pain	Improves long-term memory deficits under high task difficulty	Pain may occupy limited cognitive resources, reducing availability for non-pain-related tasks	(Saffarpour et al., 2017)
Curcumin	Male Sprague Dawley rats	Neuropathic pain	Improves memory deficits in CCI rats	Associated with enhanced hippocampal neurogenesis and synaptic plasticity	(Du et al., 2021)
Nanocurcumin	Male albino Wistar rats	Neuropathic pain	Improves spatial learning/memory deficits	Linked to reduced hippocampal IL-1β and TNF-α levels	(Saffarpour et al., 2021)
Clemastine	Adult male C57BL/6 J mice	Neuropathic pain	Improves memory deficits in CCI mice	Promotes remyelination, reverses myelin loss, and normalizes neuronal activity	(Zhu et al., 2024)

MR16-1, anti-mouse IL-6 receptor antibody; d-Asp, d-Aspartate; AIS, initial segment; SAM, S-adenosylmethionine; Res, Resveratrol; NR2B, N-Methyl-D-Aspartate Receptor Subunit 2B; GluR1, Glutamate Receptor 1; CREB, cAMP Response Element-Binding.

## 5 Conclusion and future perspectives

Accumulating clinical and preclinical evidence strongly indicates the detrimental effects of chronic pain on various cognitive domains, including memory, attention, and executive function. This review synthesizes the neurobiological mechanisms underlying cognitive dysfunction associated with chronic pain. It emphasizes the structural and functional remodeling in key brain regions, such as the hippocampus and PFC, along with their interconnected circuits. Cellular and molecular pathological changes, including neuroinflammation, impairments in synaptic plasticity, and epigenetic dysregulation, are identified as critical factors contributing to cognitive decline. Current therapeutic strategies, encompassing pharmacological agents and neuromodulation techniques, are systematically evaluated, highlighting their dual roles in alleviating pain and preserving cognitive function.

Future research should focus on three key directions to address existing knowledge gaps. First, elucidating the molecular mechanisms, particularly the spatiotemporal dynamics of epigenetic modifications such as DNA methylation and histone acetylation, will clarify their roles in pain-related memory impairment and inform targeted drug development. Second, integrating neuromodulation techniques, including transcranial stimulation and optogenetics, with microbiota-based therapies, such as probiotics and short-chain fatty acid supplementation, may synergistically enhance synaptic plasticity and neural network resilience. Third, understanding pain subtype-specific mechanisms, especially distinguishing neuropathic from inflammatory pain, is essential for developing personalized treatment strategies. Addressing translational challenges, such as optimizing blood–brain barrier penetration, ensuring long-term safety, and validating multimodal biomarkers, will require interdisciplinary collaboration. Advancements in these areas are anticipated to transform chronic pain management, shifting the focus from symptomatic relief to neuroprotective precision medicine, ultimately reducing the global burden of pain-cognition comorbidities.
